# Epidemiological Analysis of Hospitalized Children with Acute Asthma Exacerbation over 30 Years

**DOI:** 10.3390/arm94030034

**Published:** 2026-05-29

**Authors:** Xuee Zhuang, Mengyuan Liu, Kunhong Lin, Yangxin Xiao, Xuyan Zhao, Zifan Gai, Yan Xing

**Affiliations:** 1 Peking University Third Hospital, Beijing 100191, China; 10250207@hqu.edu.cn (X.Z.); 2411110550@stu.pku.edu.cn (M.L.); 2511110541@stu.pku.edu.cn (K.L.); 2311210427@stu.pku.edu.cn (Y.X.); 2411210411@stu.pku.edu.cn (X.Z.); 2511210423@stu.pku.edu.cn (Z.G.); 2 Quanzhou Maternal and Child Health Care Hospital (Quanzhou Children’s Hospital), Quanzhou 362000, China

**Keywords:** children, allergy, asthma exacerbation, epidemiology

## Abstract

**Highlights:**

**What are the main findings?**

- Over 30 years, there has been a gradual decline in pediatric hospitalizations for acute asthma exacerbations in a Tertiary Hospital in Beijing. The highest number of hospitalizations occurred in 1990 (93 patients, 8.4%), while the lowest coincided with the COVID-19 pandemic in 2020 (16 patients, 1.4%).
- Hospitalizations peaked in autumn, especially in October (13.29%), and were most common among preschool-age children (46.75%).

**What are the implications of the main findings?**

- The decline in hospitalizations suggests that childhood asthma prevention and control measures have led to improvements in asthma management.
- Further efforts are needed to enhance asthma prevention and management strategies, particularly for high-risk groups and during peak seasons, to reduce acute exacerbations and hospitalizations.

**Abstract:**

Objectives: To provide an evidence-based reference for preventing and managing pediatric acute asthma exacerbations by examining epidemiology and long-term trends of hospitalized cases in a tertiary hospital in Beijing over 30 years. Methods: Retrospective analysis of clinical data from children hospitalized for acute asthma exacerbation at Peking University Third Hospital from 1994 to 2023. Data collected included demographics, onset timing, and hospital stay duration, with distribution patterns analyzed across ages, years, seasons, and months. Results: The study included 1106 patients (65.73% male, 34.27% female) with a median age of 4 years. Hospitalizations peaked in 1999 (8.40%) and declined, reaching the lowest point in 2020 (1.45%) coinciding with the COVID-19 pandemic. Most admissions occurred in autumn (34.27%), especially in October (13.29%). The average hospital stay was 5.35 ± 2.65 days, longest for toddlers. Conclusions: Over 30 years, pediatric hospitalizations for acute asthma exacerbations in this tertiary center have shown a declining trend, suggesting improved asthma management. However, the persistent autumn peak and male predominance highlight the need for targeted prevention strategies—particularly for male and preschool-aged children before the autumn school term—to further reduce acute exacerbations and hospitalizations.

## 1. Introduction

Asthma is the most prevalent chronic respiratory condition that is not caused by infection in childhood. Over the past three decades, rapid industrialization has significantly altered lifestyles and living environments, contributing to a notable increase in childhood asthma prevalence nationwide. Meanwhile, diagnostic and management standards have also improved markedly. Consistent with global epidemiological trends, childhood asthma prevalence has increased steadily both domestically and internationally [1,2]. Asthma exerts multifaceted adverse effects on pediatric health, compromising physical growth and development, impairing psychological well-being, and imposing substantial economic burdens on families and society at large [3].

The pathogenesis of bronchial asthma is a highly complex process; increasing evidence indicates that asthma results from the combined effects of genetic and environmental factors [4]. Acute exacerbations of asthma are the leading reason for hospital admissions among children with this condition, with approximately 17% of pediatric patients with asthma requiring inpatient management [5]. Many risk factors can cause acute exacerbation of asthma, including allergens, viral infections, pollutants, tobacco smoke, and medications. Reducing children’s exposure to risk factors can improve asthma control and decrease the need for therapeutic medications. However, environmental conditions, climate, and demographic characteristics vary significantly by region. As the capital of China, Beijing faces unique geographical challenges, including high air pollution, which makes epidemiological analysis of asthma in this region particularly important. Therefore, assessing the etiological factors underlying acute exacerbation of asthma and reducing the hospitalization rate related to such episodes are critical for lowering the morbidity and mortality of childhood asthma.

Based on this, this study conducted a retrospective analysis of the clinical data of children aged 1–15 years who were hospitalized due to acute asthma exacerbation in a tertiary hospital in Beijing from 1994 to 2023. The primary goal was to investigate the clinical characteristics of these patients and to provide evidence-based resources for the prevention, treatment, and long-term management of acute asthma exacerbation in childhood in Beijing. The findings are reported as follows.

## 2. Materials and Methods

### 2.1. General Information

This study included 1106 children aged 1–15 years with acute asthma exacerbation admitted from January 1994 to December 2023 to the Department of Pediatrics, Peking University Third Hospital. Based on age, the patients were stratified into the following three groups: toddlerhood (1 ≤ age < 3 years), preschool age (3 ≤ age < 7 years), and school age (7 ≤ age < 15 years).

It was infeasible to conduct informed consent notification and obtain signed informed consent forms, as this study is a retrospective analysis based on clinical medical record data with an extended time span. The need for signing relevant informed consent forms was waived on the premise of maximizing the protection of patients’ rights, interests, and privacy, following submission to and review by the Medical Ethics Committee of Peking University Third Hospital. Ethics Approval Number: M20250542.

### 2.2. Inclusion and Exclusion Criteria

Inclusion criteria were as follows: (1) Pediatric inpatients who met the diagnostic criteria for childhood asthma issued in China across different eras [6,7,8,9,10]; (2) those aged 1 to 15 years old (inclusive of 1 and 15 years old); and (3) those with complete medical records.

Exclusion criteria were as follows: (1) Children with asthma in the clinical remission or chronic persistent phase; (2) children who have enrolled multiple times during the same acute exacerbation phase; (3) those with specific respiratory system diseases such as bronchial foreign bodies, respiratory tract malformations, lung tumors, or lung trauma; and (4) those with severe diseases involving other systems including the heart, brain, liver, gastrointestinal tract, and kidneys.

### 2.3. Methods

This study analyzed demographic (age, gender, date of birth) and clinical data (visit year, visit season, visit month, length of hospital stay) from 1994 to 2023 of hospitalized children aged 1–15 years with acute asthma exacerbation. The gender distribution of the children was specifically examined, and the distribution characteristics by different years, seasons, months, and lengths of hospital stay were also assessed.

### 2.4. Statistical Methods

SPSS 24.0 software was used for statistical data analysis. Descriptive analysis was employed to examine the general demographic characteristics of hospitalized children with acute asthma exacerbation. Measurement data conforming to normal distribution were expressed as mean ± standard deviation ($\bar{x}$ ± SD); for those not conforming to normal distribution, median and interquartile range were used. T-test and one-way analysis of variance (ANOVA) were commonly used for assessing measurement data, while chi-square test (χ^2^ test) and rank sum test were used for analyzing count data. The statistical significance was set at *p* < 0.05.

## 3. Results

### 3.1. Gender Distribution of Hospitalized Children with Acute Asthma Exacerbation

This study enrolled 1106 hospitalized children with acute asthma exacerbation, consisting of 727 males (65.73%) and 379 females (34.27%).

### 3.2. Age (Group) Distribution of Hospitalized Children with Acute Asthma Exacerbation

The median age of hospitalized children with acute asthma exacerbation in this study was 4 years (interquartile range, IQR: 2–7). The participants were stratified into the following three age groups: toddlerhood (1 ≤ age < 3 years), preschool age (3 ≤ age < 7 years), and school age (7 ≤ age < 15 years), with 306, 517, and 283 cases, respectively. The preschool age group accounted for approximately half of the total cohort, which is the highest proportion (46.75%), followed by the toddlerhood group, which constituted more than a quarter (27.67%) of the patients.

### 3.3. Gender Differences in Hospitalized Children with Acute Asthma Exacerbation Across Different Age Groups

**Figure 1:**
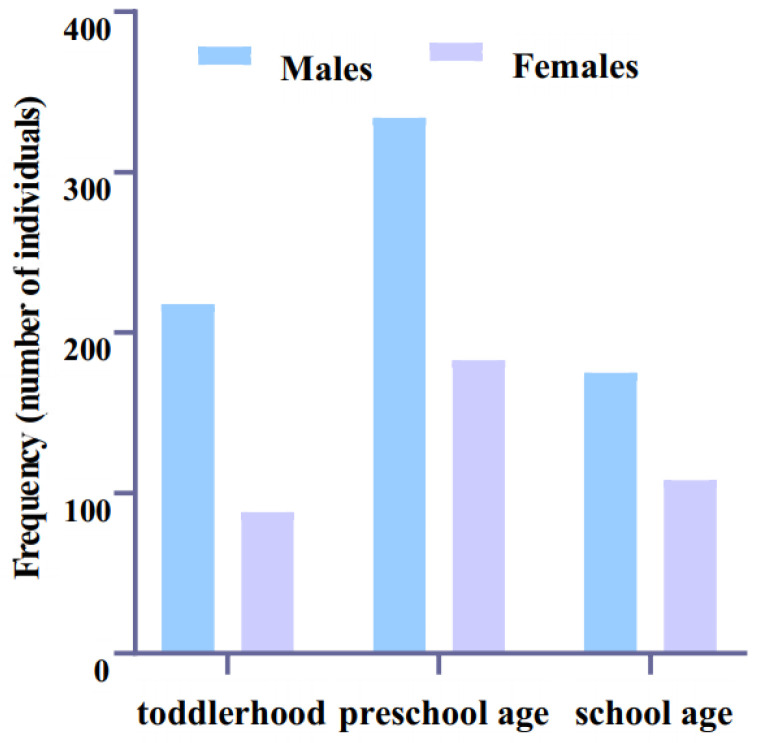
Gender disparities in hospitalized children with acute asthma exacerbation among different age groups.

The number of male hospitalized children with acute asthma exacerbation was consistently higher than that of females across all age groups. Specifically, the male-to-female ratio was approximately 2.5:1 (218 vs. 88), 1.8:1 (334 vs. 183), and 1.6:1 (175 vs. 108) in the toddlerhood, preschool age, and school age groups, respectively. Figure 1 illustrates these gender disparities across age strata.

Males were more predominant than females across all age groups, with gender disparities diminishing with increasing age. A significant difference in gender distribution among the three groups (χ^2^ = 6.322, *p* < 0.05; Table 1) was exhibited through statistical analysis. Post hoc pairwise comparisons showed no significant differences between toddlerhood and preschool age (χ^2^ = 3.836, *p* = 0.05) or between preschool and school age (χ^2^ = 0.605, *p* = 0.437). However, a remarkable difference was noted between toddlerhood and school age (χ^2^ = 5.856, *p* = 0.016).

### 3.4. Annual Distribution of Hospitalized Children with Acute Asthma Exacerbation

**Figure 2:**
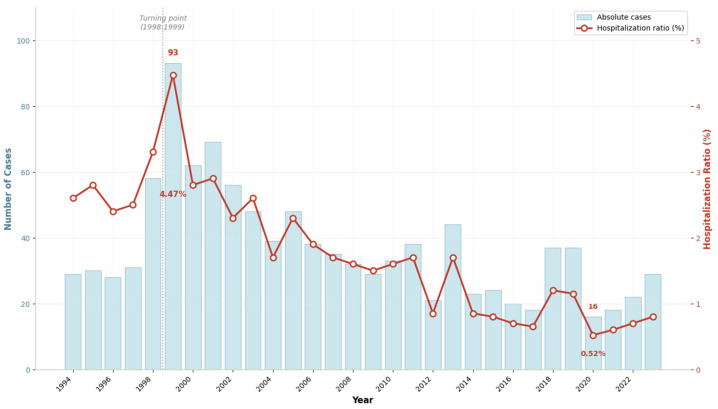
Annual pediatric asthma admissions and hospitalization ratios at Peking University Third Hospital (1994–2023).

From 1994 to 2023, 1106 children with acute asthma exacerbation were admitted to Peking University Third Hospital. The asthma hospitalization ratio (asthma cases/total pediatric discharges) peaked in 1999 at 4.47% (93 cases), the highest proportion in the 30-year series (1.74% ± 0.96%). Between 1994 and 1997, both absolute admissions and hospitalization ratios remained relatively stable, followed by a significant increase in 1998 and the 1999 peak. After 2000, the hospitalization ratio showed a significant downward trend, with two minor peaks in 2013 (1.56%) and 2018–2019 (1.10–1.14%). In 2020, admissions dropped to their lowest level (16 cases, 0.78%), coinciding with the COVID-19 pandemic, followed by a slow recovery through 2023 (29 cases, 0.75%). The annual admission figures and hospitalization ratios are illustrated in Figure 2.

### 3.5. Monthly Variation in Admissions

**Figure 3:**
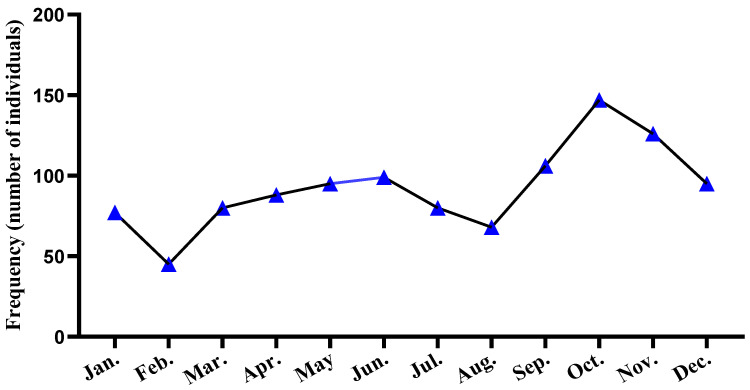
Monthly distribution of hospitalized children with acute asthma exacerbation.

Figure 3 illustrates the monthly distribution of hospitalized children with acute asthma exacerbation at Peking University Third Hospital between 1994 and 2023. The highest monthly admission volume was in October (147 cases, 13.29%), followed by November (126 cases, 11.39%) and September (106 cases, 9.58%). In contrast, February had the lowest reported number of admissions (45 cases, 4.06%).

### 3.6. Seasonal Patterns of Admissions

**Figure 4:**
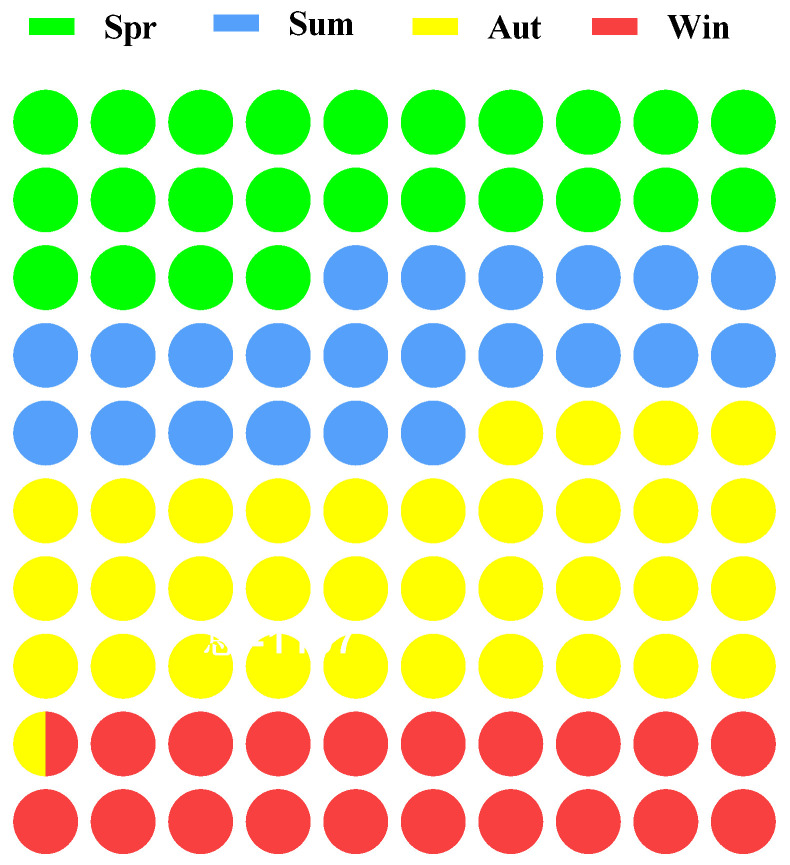
Seasonal distribution of hospitalized children with acute asthma exacerbation.

Hospitalized children admitted to Peking University Third Hospital between 1994 and 2023 owing to acute asthma exacerbation were categorized according to season based on meteorological standards as follows: spring (March–May), summer (June–August), autumn (September–November), and winter (December–February of the following year). As illustrated in Figure 4, autumn was consistently the peak season for admissions. The number of hospitalized patients in each season was as follows: spring, 263 cases (23.78%); summer, 247 cases (22.33%); autumn, 379 cases (34.27%); and winter, 217 cases (19.62%).

### 3.7. Age-Related Seasonal Patterns

**Figure 5:**
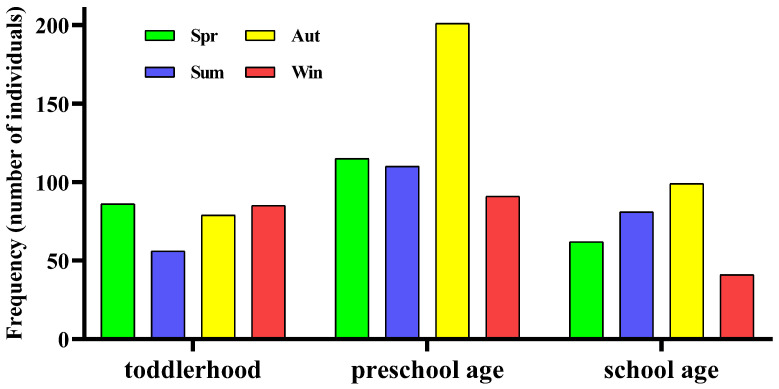
Seasonal distribution of hospitalized children with acute asthma exacerbation across different age groups.

The seasonal distribution of hospitalized children with acute asthma exacerbation varied across age groups. Preschool and school-aged children consistently demonstrated an autumn peak in admissions, as illustrated in Figure 5, whereas toddlerhood children demonstrated a higher admission volume in spring and winter.

### 3.8. Gender Differences in Seasonal Distribution

**Figure 6:**
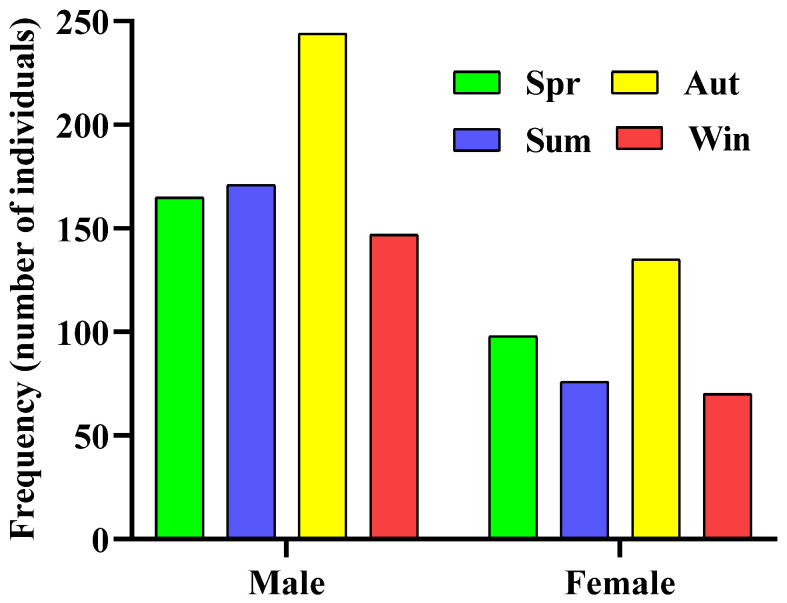
Seasonal distribution of hospitalized children with acute asthma exacerbation by gender.

Both genders demonstrated an autumn peak in the seasonal distribution of hospitalized cases, as depicted in Figure 6.

### 3.9. Length of Hospital Stay in Children with Acute Asthma Exacerbation

**Figure 7:**
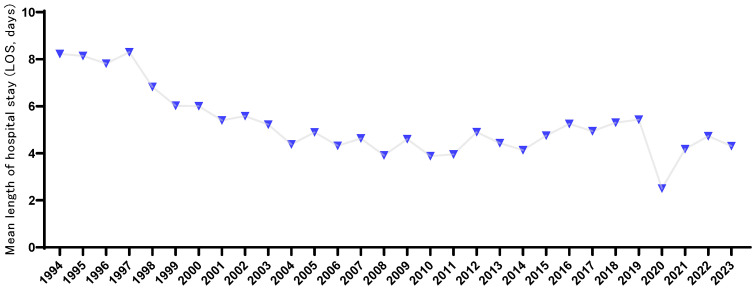
Annual distribution of mean Length of Hospital Stay (LOS) for acute asthma exacerbation.

The mean length of hospital stay (LOS) for children admitted with acute asthma exacerbation was 5.35 ± 2.65 days over the 30 years from 1994 to 2023. The overall trend showed a downward decline, followed by a stable plateau from 2004 onward, with the lowest LOS documented in 2020 (Figure 7).

### 3.10. Length of Hospital Stay (LOS) for Acute Asthma Exacerbation Across Different Age Groups

**Figure 8:**
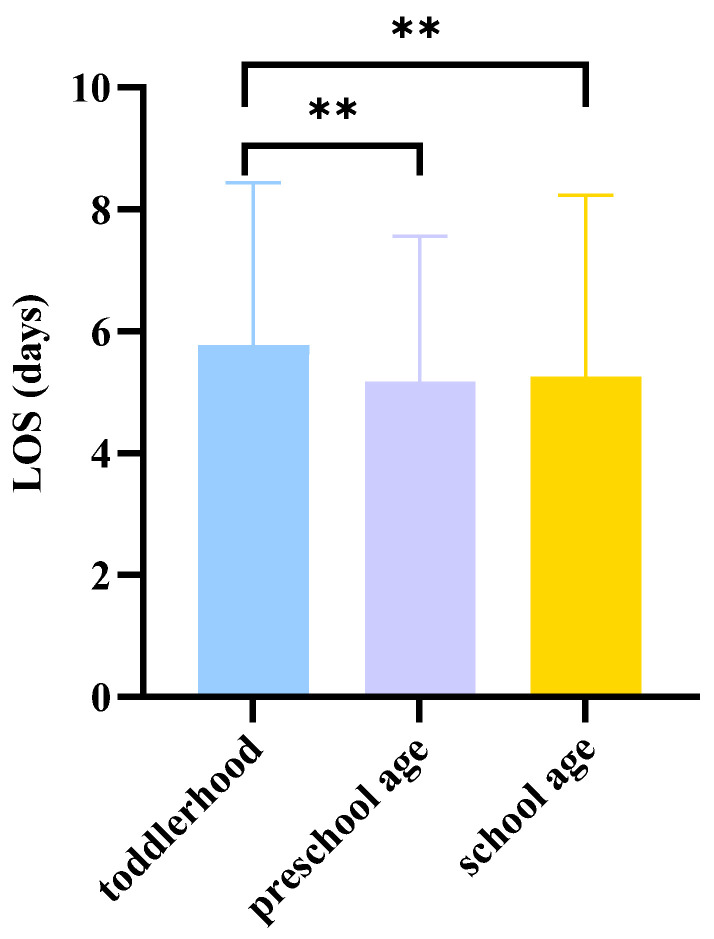
Differences in the Length of Hospital Stay (LOS) among hospitalized children with acute asthma exacerbation across age groups. ** *p* < 0.05.

There was a statistically significant difference in LOS among hospitalized children with acute asthma exacerbation across distinct age groups (*F* = 5.20, *p* = 0.006). The toddlers demonstrated the longest duration of hospitalization. Post hoc pairwise comparisons (LSD) showed that toddlers had a significantly longer LOS than both preschool children (MD = 0.60 days, 95% CI: 0.22 to 0.97, *p* = 0.002) and school-age children (mean difference = 0.52 days, 95% CI: 0.09 to 0.95, *p* = 0.018). In contrast, there was no statistically significant difference in LOS between preschool and school-age children (mean difference = −0.08 days, 95% CI: −0.46 to 0.31, *p* = 0.689). These results are illustrated in Figure 8.

### 3.11. Length of Hospital Stay (LOS) for Acute Asthma Exacerbation by Gender

**Figure 9:**
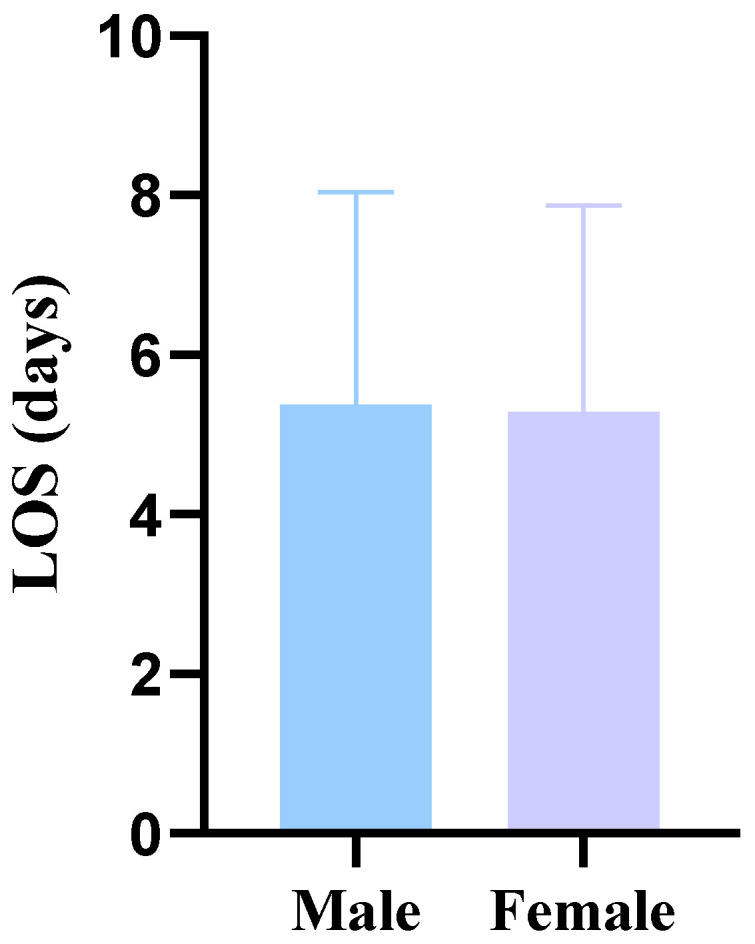
Differences in Length of Hospital Stay (LOS) among hospitalized children with acute asthma exacerbation by gender.

There was no statistically significant difference in LOS between males and females among hospitalized children with acute asthma exacerbation (males: 5.38 ± 2.66 days; females: 5.29 ± 2.58 days; *F* = 0.95, *p* = 0.58) (Figure 9).

## 4. Discussion

Acute asthma exacerbations are a common cause of pediatric hospitalization [11], yet their long-term epidemiological patterns remain incompletely characterized. In this 30-year (1994–2023) retrospective analysis of clinical data from a tertiary hospital in Beijing, we identified age-specific seasonal patterns of pediatric hospitalizations for acute asthma exacerbations: preschool and school-aged children exhibited an autumn peak, whereas toddlers showed a predominance of exacerbations in spring and winter. This finding suggests that the dominant triggers of asthma exacerbations may differ across age groups—post-enrollment cross-infections may be a key driver in school-aged children, while respiratory viral infections may play a larger role in toddlers. To our knowledge, this is the first 30-year retrospective analysis of pediatric hospitalization trends for acute asthma exacerbations specifically in Beijing, providing long-term epidemiological evidence for this megacity with distinct seasonal air pollution patterns. The sample size of this study was relatively modest; nevertheless, the 30-year follow-up provides valuable longitudinal data for revealing these seasonal and age-specific patterns.

This study found that male children represented a higher proportion of hospitalizations than females, with a male-to-female ratio of approximately 1.9:1, supporting results from earlier studies [12]. This male predominance is consistent with the overall incidence pattern of childhood asthma, where boys are persistently more frequently affected than girls [13]. Notably, this gender disparity gradually narrowed with age: the male-to-female ratios were approximately 2.5:1, 1.8:1, and 1.6:1 in toddlers, preschoolers, and school-aged children, respectively. This pattern aligns with the observed reversal of gender distribution in asthma epidemiology—asthma is more prevalent in young boys, whereas adult women exhibit a higher prevalence—suggesting that hormonal factors may play a role [14,15].

Asthma can occur throughout life but is especially common in childhood. Epidemiological studies show that 87.6% of children with asthma experience their first acute exacerbation by age 5 years [16]. This study revealed that the average age of children hospitalized for acute asthma exacerbations was 4 years. Preschool children represented the largest proportion (46.75%), followed by toddlers (27.67%), which is in accordance with the evidence reported in existing literature [17]. In China, kindergarten enrollment typically begins at age 3. Upon entering kindergarten, children may struggle to adjust to the new environment and are at higher risk for cross-infections—respiratory tract infections are widely recognized as a key precipitating factor for asthma exacerbations. Importantly, the clinical manifestations of asthma in young children are often non-specific. Moreover, many children with wheezing before 3 years discontinue wheezing episodes after 6 years, leading to the consensus that bronchial asthma should not be diagnosed prematurely in this young population [18]. However, our finding of higher hospitalization rates in younger children underscores the urgent need for early accurate diagnosis and timely intervention to prevent exacerbations and optimize long-term clinical outcomes.

The age-specific seasonal pattern was further reflected in monthly admission data. In this 30-year retrospective analysis of a tertiary hospital in Beijing, pediatric asthma admissions peaked in October (147 cases, 13.29%), followed by November (126 cases, 11.39%) and September (106 cases, 9.6%), with the lowest rate in February (45 cases, 4.1%). Multiple biological and environmental factors may contribute to the autumn peak, including elevated ambient pollen concentrations [19], increased proliferation of respiratory pathogens [20], and seasonal fluctuation of indoor dust mite density reaching its annual maximum [21]. As the capital of China with high vehicle ownership, Beijing experiences significantly elevated traffic-related air pollution (TRAP) during the heating season under unfavorable meteorological dispersion conditions, which may synergize with biological factors. Recent studies have revealed that NO_2_ and PM_2.5_ damage airway epithelium through oxidative stress-mediated TRPV1 pathway activation, while large diurnal temperature variations and humidity fluctuations in autumn further amplify this effect; concurrently, PM_2.5_ co-exposure with temperature fluctuations disrupts epithelial barrier function [22,23,24]. Epidemiological studies have also confirmed that the synchronous correlation between autumn PM_10_ and asthma exacerbations is significantly stronger than in other seasons [25]. These mechanisms corroborate our clinical observations: the October admission peak coincides with the superposition of heating season onset, pollen exposure, pathogen proliferation, and increased cross-infections following school enrollment, creating a multi-hit scenario. While the 2020 decline coincided with the COVID-19 pandemic, this likely reflects both reduced viral transmission from public health measures and altered healthcare-seeking behavior. A study from Chengdu similarly reported a significant drop in monthly asthma cases in February 2020, with higher rates of severe attacks (9.69%) and reduced pulmonary function testing (34.5%), suggesting that milder cases may have avoided hospital care while more severe cases still presented [26]. Additionally, research on rhinovirus (RV) transmission demonstrated that social distancing and school closures markedly reduced respiratory viral infections and asthma healthcare utilization, though RV resurged specifically after school reopening due to its unique resistance to masking and disinfectants. These findings collectively suggest that socially driven factors, including viral transmission dynamics and school policies, played a pivotal role in shaping pediatric asthma hospitalization patterns during the pandemic [27].

The age-specific seasonal pattern we observed suggests that the dominant triggers of asthma exacerbations may differ across age groups. The autumn peak in school-aged and preschool children is consistent with the hypothesis of increased cross-infections following school enrollment. The spring/winter peak in toddlers, by contrast, aligns with the typical seasonality of viral respiratory infections (e.g., RSV, influenza). Although this study lacks direct viral infection data to confirm this mechanism, the age-specific seasonal pattern provides indirect but compelling evidence for trigger-specific hypotheses. Notably, southern regions such as Shanghai, with their subtropical climate and higher humidity, may exhibit distinct seasonal distributions of asthma exacerbations [28]. Therefore, our single-center findings from Beijing may not represent national trends, and multi-center studies across diverse climatic zones in China are needed to understand regional variations. Understanding these marked seasonal variations is essential for enhancing asthma prevention and control strategies. Our findings suggest age-specific targeted preventive measures: for preschool and school-aged children, interventions (e.g., influenza vaccination, reinforcement of asthma action plans) should be implemented before August each year (prior to school reopening); for toddlers, preventive efforts should focus on the winter viral season (e.g., RSV prophylaxis, hand hygiene campaigns). These age-specific, season-specific strategies may inform asthma control policies in Beijing and climatically similar regions, pending validation through future studies with trigger-specific data.

This study was limited by the sole analysis of hospitalization data for acute asthma exacerbations in children, with single-center data that may not reflect national trends or primary/secondary care settings. On the other hand, due to the 30-year retrospective design, complete clinical information was unavailable. Future studies should expand the collection of data to include the incidence of childhood asthma, specific triggers of acute exacerbation episodes, age at the initial onset, treatment modalities, and clinical outcomes, and should incorporate multicenter designs across diverse regions to validate these findings. Such comprehensive analyses will offer more robust data support for the prevention and management of acute asthma exacerbations in children.

## 5. Conclusions

In conclusion, male patients outnumbered female patients among children aged 1–15 years hospitalized for acute asthma exacerbations in this tertiary center. Preschool children comprised the largest proportion of admissions, with the highest hospitalization rate occurring in autumn and a distinct peak in October. Comprehensive prevention and control strategies should be customized to reduce the incidence and hospitalization rate of childhood acute asthma exacerbations, based on the environmental characteristics of Beijing.

## Figures and Tables

**Table 1:** Comparison of gender disparities in hospitalized children with acute asthma exacerbation across different age groups.

Group	Total (*n*)	Males	Females	*χ*^2^ Test	
				*χ*^2^ Value	*p* Value
toddlerhood	306	218 (71.24%)	88 (28.76%)	6.32	0.04
preschool age	517	334 (64.60%)	183 (35.40%)		
school age	283	175 (61.84%)	108 (38.16%)		

## Data Availability

The original contributions presented in this study are included in the article. Further inquiries can be directed to the corresponding authors.

## Abbreviations

The following abbreviations are used in this manuscript:

LOS	Length of Hospital Stay
ANOVA	One-way analysis of variance
IQR	Interquartile range
MD	Mean Difference
TRAP	Traffic-Related Air Pollution
NO_2_	Nitrogen Dioxide
PM_2.__5_	Particulate Matter ≤ 2.5 micrometers
TRPV1	Transient Receptor Potential Vanilloid 1
RV	rhinovirus
